# Histone acetyltransferase 1 promotes gemcitabine resistance by regulating the PVT1/EZH2 complex in pancreatic cancer

**DOI:** 10.1038/s41419-021-04118-4

**Published:** 2021-09-25

**Authors:** Yan Sun, Dianyun Ren, Yingke Zhou, Jian Shen, Heshui Wu, Xin Jin

**Affiliations:** 1 Department of Pancreatic Surgery, Union Hospital, Tongji Medical College, Huazhong University of Science and Technology, 430022 Wuhan, China; 2 Sino-German Laboratory of Personalized Medicine for Pancreatic Cancer, Union Hospital, Tongji Medical College, Huazhong University of Science and Technology, 430022 Wuhan, China; 3 Department of Urology, The Second Xiangya Hospital, Central South University, 410011 Changsha, Hunan China; 4 Uro-Oncology Institute of Central South University, 410011 Changsha, Hunan China

**Keywords:** Cancer therapy, Oncogenes

## Abstract

The poor prognosis of pancreatic cancer is primarily due to the development of resistance to therapies, including gemcitabine. The long noncoding RNA PVT1 (lncRNA PVT1) has been shown to interact with enhancer of zeste 2 polycomb repressive complex 2 subunit (EZH2), promoting gemcitabine resistance in pancreatic cancer. In this study, we found histone acetyltransferase 1 (HAT1) enhanced the tolerance of pancreatic cancer cells to gemcitabine and HAT1-mediated resistance mechanisms were regulated by PVT1 and EZH2. Our results showed that the aberrant HAT1 expression promoted gemcitabine resistance, while silencing HAT1 restored gemcitabine sensitivity. Moreover, HAT1 depletion caused a notable increase of gemcitabine sensitivity in gemcitabine-resistant pancreatic cancer cell lines. Further research found that HAT1 increased PVT1 expression to induce gemcitabine resistance, which enhanced the binding of bromodomain containing 4 (BRD4) to the PVT1 promoter, thereby promoting PVT1 transcription. Besides, HAT1 prevented EZH2 degradation by interfering with ubiquitin protein ligase E3 component n-recognin 4 (UBR4) binding to the N-terminal domain of EZH2, thus maintaining EZH2 protein stability to elevate the level of EZH2 protein, which also promoted HAT1-mediated gemcitabine resistance. These results suggested that HAT1 induced gemcitabine resistance of pancreatic cancer cells through regulating PVT1/EZH2 complex. Given this, Chitosan (CS)-tripolyphosphate (TPP)-siHAT1 nanoparticles were developed to block HAT1 expression and improve the antitumor effect of gemcitabine. The results showed that CS-TPP-siHAT1 nanoparticles augmented the antitumor effects of gemcitabine in vitro and in vivo. In conclusion, HAT1-targeted therapy can improve observably gemcitabine sensitivity of pancreatic cancer cells. HAT1 is a promising therapeutic target for pancreatic cancer.

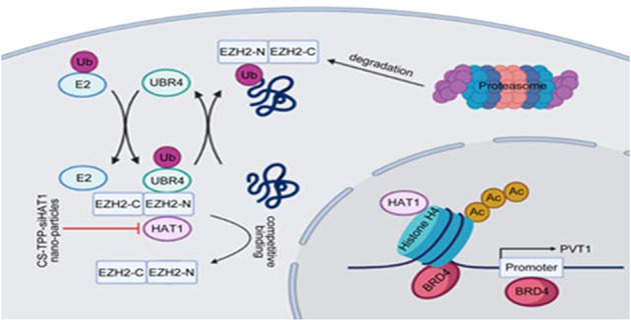

## Introduction

Pancreatic cancer is a particularly aggressive and lethal malignancy of the digestive system [1, 2]. Early diagnosis of pancreatic carcinoma is challenging due to its anatomical position; hence, only 15% of pancreatic cancer patients undergo surgical resection [3, 4]. Pancreatic cancer has a poor prognosis and extremely high mortality and morbidity rates, with a 5-year survival rate of less than 5% [5, 6].

Chemotherapy with gemcitabine is the treatment of choice for pancreatic cancer patients who were not eligible for surgery [7]. Gemcitabine inhibits pancreatic cancer cell proliferation by replacing cytidine during DNA replication and blocking the biosynthesis of deoxyribonucleotides [8]. However, the development of resistance to gemcitabine is not uncommon among pancreatic cancer patients undergoing treatment [9]. Drug resistance can be internal (innate resistance) or acquired (acquired resistance) after multiple treatment cycles [10]. Findings from large-scale technologies, including proteomics and next-generation RNA sequencing, suggest that numerous proteins mediate gemcitabine resistance [11]. For instance, aberrant expression of enhancer of zeste homolog 2 (EZH2) in pancreatic cancer cells has been linked to gemcitabine resistance, possibly due to the downregulation of the tumor suppressor p27^Kip1^ [12]. Additionally, silencing of the long noncoding RNA (lncRNA) PVT1 increased gemcitabine sensitivity in pancreatic cancer cells [13, 14]. Interestingly, PVT1 has been found to form a complex with EZH2, a key step in the development of gemcitabine resistance in pancreatic cancer [14].

We have previously shown that aberrant expression of histone acetyltransferase 1 (HAT1) enhanced PD-L1 expression and promoted pancreatic cancer cell proliferation by modulating the function of BRD4. Herein, we show that HAT1 knockdown in pancreatic cancer cells increases gemcitabine sensitivity and decreases PVT1/EZH2 complex levels, suggesting that HAT1 may represent a promising therapeutic target in pancreatic cancer.

## Results

### Aberrant HAT1 expression promotes gemcitabine resistance in pancreatic cancer cells

**Fig. 1:**
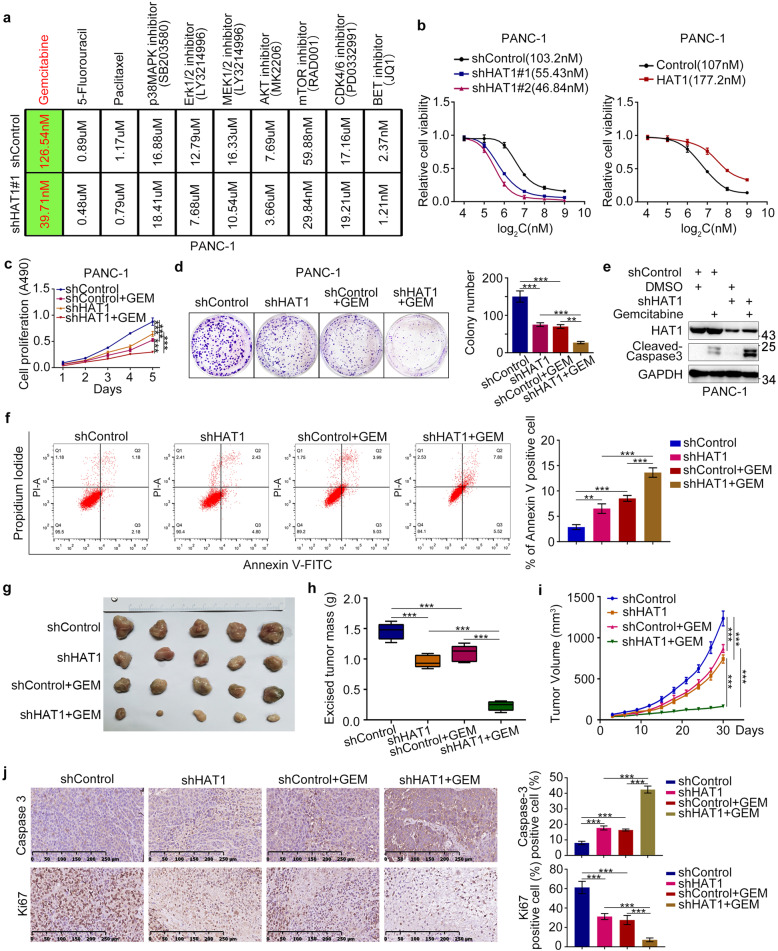
Aberrant HAT1 expression promotes gemcitabine resistance in pancreatic cancer cells. **a** MTS assay was used to detect the viability of PANC-1 after treating with different drugs. GraphPad Prism 7.0 software was used to calculate IC50. **b** MTS assay detected the viability of PANC-1 cells with normal, knockdown, and overexpressing HAT1 after treatment with gemcitabine. The cell viability curve showed IC50 values of gemcitabine among different groups. **c–f** PANC-1 cells were infected with lentivirus expressing control or HAT1-specific shRNAs. After infecting 72 h, cells were harvested and treated with gemcitabine (50 nM) for MTS assay (**c**), colony formation assay (**d**), cleaved caspase-3 activity assay (**e**), and Annexin-V/Propidium Iodide assay (**f**). All data were shown as mean values ± SD (*n* = 3). **P < 0.01; ***P < 0.001. **g**–**j** PANC-1 cells were transfected with indicated plasmids for 72 h. Cell were subcutanousely injected into the nude mice. These mice were injected intraperitoneally with or without gemcitabine (10 mg/kg) every 3 days when tumor volume reached to 50 mm^3^. The tumor volume was measured every 3 days and all tumors were harvested for photograph and weight after 30 days (**g–i**). All tumors were conducted caspase-3 and Ki67 analysis by IHC staining (**j**). Data presented as Means ± SD (*n* = 5). ***P < 0.001.

HAT1 is often upregulated in pancreatic cancer and promotes pancreatic cancer cell proliferation by regulating PD-L1 expression; however, the oncogenic role of HAT1 in pancreatic cancer remains poorly understood. Several studies had found that the expression of histone acetyltransferase (HAT) genes was related to drug resistance [15–17]. As is well known, pancreatic cancer was easily tolerated by chemotherapy, while upregulated HAT1 might led to the results. Thus, we explored the influence of abnormal HAT1 expression on the sensitivity of several commonly used drugs in pancreatic cancer cells, which showed the significantly reduced IC50 for gemcitabine when HAT1 was knocked down, while the sensitivity of other drugs was slightly reduced or unchanged (Fig. 1a). Furtherly, HAT1 silencing increased three pancreatic cancer cell lines sensitivity to gemcitabine; HAT1 overexpression had the opposite effect (Fig. 1b and Fig. S1a, b). The expression of HAT1 also was upregulated obviously in pancreatic cancer cells compared to the normal cells (Fig. S1c), which might be the potential cause of gemcitabine resistance. Additionally, MTS and colony formation assays revealed that HAT1-knockdown cells grew slower than control cells in the presence of gemcitabine (Fig. 1c, d and Fig. S1d, e). Moreover, HAT1 silencing in pancreatic cancer cells enhanced apoptosis in response to gemcitabine treatment (Fig. 1e, f and Fig. S1f). Consistently, HAT1 knockdown enhanced the tumor-suppressive effects of gemcitabine in tumor xenografts (Fig. 1g). Specifically, tumors with HAT1 knockdown exhibited the slowest tumor growth, the highest caspase-3 levels, and the lowest Ki67 levels in response to gemcitabine treatment (Figs. 1h–j).

Besides, we constructed the gemcitabine-resistant PANC-1. The cell morphology changed significantly and the sensitivity reduced by 7.79 times compared to ordinary PANC-1 (Fig. S1g and S1h). GR-PANC-1 was also used to carry out MTS, clone formation and caspase-3 activity detecting (Fig. S1i–k). The results showed that silencing HAT1 could recover the gemcitabine sensitivity. Moreover, the expression of HAT1 increased in GR-PANC-1, which further illustrated HAT1 promoted the gemcitabine resistance of pancreatic cancer cells (Fig. S1l). These findings suggest that aberrant HAT1 expression affects the response of pancreatic cancer cells to gemcitabine in vitro and in vivo.

### HAT1 enhances PVT1 expression by facilitating BRD4 binding to PVT1 promoter to promote gemcitabine resistance

**Fig. 2:**
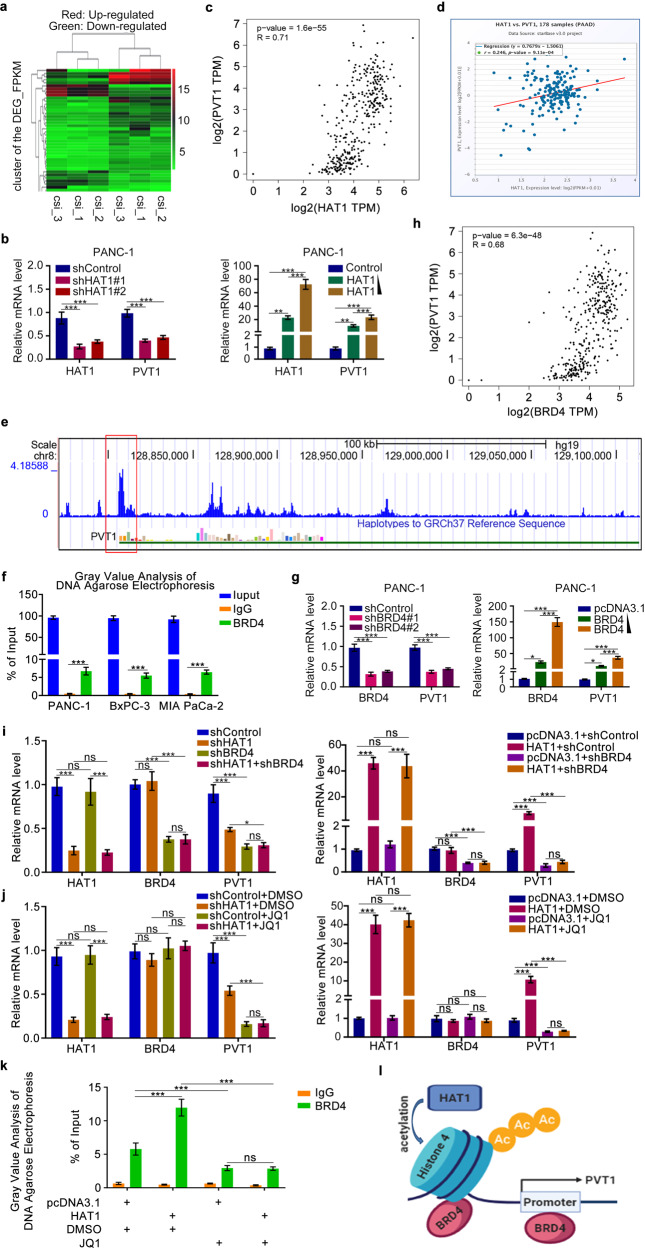
HAT1 enhances PVT1 expression by facilitating BRD4 binding to PVT1 promoter to promote gemcitabine resistance. **a** Heatmap showed the differential expressed genes of PANC-1 cells infected by shControl or shHAT1. Red, upregulated genes; Green, downregulated genes. **b** PANC-1 cells were infected with lentivirus expressing control, HAT1-specific shRNAs, pcDNA3.1, or HAT1 plasmid. After infecting 48 h, cells were harvested to conduct RT-qPCR analysis. Statistical analyses were performed with one-way ANOVA followed by Tukey’s multiple comparison’s tests. All data were shown as mean values ± SD (*n* = 3). **P < 0.01; ***P < 0.001. **c,** **d** The correlation analysis between HAT1 and PVT1 based on GEPIA web (**c**) and ENCORI Pan-Can**c**er Analysis Platform (**d**). **e** UCSC Genome Browser screenshots of the BRD4 ChIP-Sequence profiles at the PVT1 gene locus in C4–2 cells reported previously. **f** PANC-1, BxPC-3, and MIA-PaCa-2 cells were treated according to the protocol of ChIP experiment, and the resulting DNA sample was subjected to DNA agarose gel electrophoresis. All data were shown as mean values ± SD (*n* = 3). ***P < 0.001. **g** PANC-1 cells were infected with lentivirus expressing control, BRD4-specific shRNAs, and BRD4 overexpressing plasmid. After infecting 48 h, cells were harvested to conduct RT-qPCR analysis. All data were shown as mean values ± SD (*n* = 3). *P < 0.05; ***P < 0.001. **h** The correlation analysis between BRD4 and PVT1 based on GEPIA web. **i** PANC-1 cells were infected with lentivirus vectors expressing control, HAT1-specific shRNAs, BRD4-specific shRNAs, pcDNA3.1, or HAT1 plasmid. After 48 h infection, cells were harvested for RT-qPCR analysis. The data shown were the mean values ± SD (*n* = 3). ns not significant; *P < 0.05; ***P < 0.001. **j** PANC-1 cells were infected with lentivirus vectors expressing control, HAT1-specific shRNAs, pcDNA3.1, or HAT1 plasmid. After 48 h infection, the cells were treated with or without JQ1 (10 μM) for another 24 h. Cells were harvested for RT-qPCR analysis. The data shown were the mean values ± SD (*n* = 3). ns not significant; ***P < 0.001. **k** PANC-1 cells were infected with pcDNA3.1 or HAT1 plasmid. After 48 h infection, the cells were treated with or without JQ1 (10 μM) for another 24 h. The cells were harvested for ChIP-qPCR analysis and DNA agarose gel electrophoresis. The data shown were the mean values ± SD (*n* = 3). ns not significant; ***P < 0.001. **l** The schematic diagram of HAT1 regulating PVT1.

Despite evidence of the critical role of HAT1 in pancreatic cancer progression and gemcitabine resistance, the underlying mechanism remains elusive. RNA sequencing of HAT1 knockdown and control PANC-1 cells yielded 945 differentially expressed genes, including 490 upregulated and 455 downregulated genes (Fig. 2a and Fig. S2a). A significant portion of these genes was directly or indirectly related to cancer (Fig. S2b). Importantly, the lncRNA PVT1, a potential downstream target gene of HAT1, was downregulated upon HAT1 silencing, whereas HAT1-overexpressing cells exhibited elevated PVT1 levels (Fig. 2b and Fig. S2b, c). Furthermore, analyses using the GEPIA tool and ENCORI Pan-Cancer Analysis Platform indicated a positive correlation between HAT1 and PVT1 expression levels (Fig. 2c, d), suggesting that HAT1 regulates PVT1 expression in pancreatic cancer cells. A previous genome-wide screen identified PVT1 as a regulator of gemcitabine sensitivity in pancreatic cancer cells [13]. MTS assay confirmed that PVT1 was a drug-related resistant gene (Fig. S2d). To verify the relevance of PVT1 in HAT1-mediated gemcitabine resistance, we performed MTS assay and found that cells with HAT1 silencing and PVT1 overexpressing proliferated significantly faster than HAT1-knockdown cells (Fig. S2e). Thus, HAT1 promotes gemcitabine resistance by enhancing PVT1 expression.

Although our findings demonstrate that HAT1 promotes PVT1 transcription, the underlying molecular mechanism is poorly understood. HAT1 could catalyze H4 acetylation (Fig. S3a, b), which is essential for the binding of the transcription activator BRD4 to histone H4 [18]. Besides, we have previously shown that HAT1 promoted PD-L1 expression in pancreatic cancer cells in a BRD4-dependent manner. We analyzed available ChIP-seq data of BRD4 [19] and identified a BRD4-binding peak in the promoter of PVT1 (Fig. 2e). This result was confirmed in pancreatic cancer cells by ChIP-qPCR (Fig. 2f). Moreover, PVT1 expression levels were decreased or increased after BRD4 silencing or overexpression, respectively (Fig. 2g and Fig. S3c), suggesting that BRD4 may regulate the transcription of PVT1. Furthermore, analysis using GEPIA indicated a strong positive correlation between BRD4 and PVT1 levels in pancreatic cancer specimens (Fig. 2h). To assess the relevance of BRD4 in the HAT1-mediated regulation of PVT1 expression, we silenced BRD4 in combination with HAT1 knockdown or HAT1 overexpression. Interestingly, BRD4 silencing attenuated the ability of HAT1 to regulate PVT1 expression (Fig. 2i). Similarly, treatment with the BRD4 inhibitor JQ1 abrogated the ability of HAT1 silencing or overexpression to regulate PVT1 expression levels (Fig. 2j). The ability of BRD4 to bind to the PVT1 promoter was also significantly reduced upon JQ1 treatment, even in HAT1-overexpressing cells (Fig. 2k). Besides, the therapeutic effects of gemcitabine were significantly enhanced whether using JQ1 to inhibit BRD4 function or directly knocking down BRD4 (Fig. S3d, e), which further indicated that BRD4 could regulate the expression of PVT1. Collectively, these data suggest that the ability of HAT1 to induce PVT1 expression in pancreatic cancer cells requires BRD4 (Fig. 2l).

### HAT1 stabilizes EZH2 to promote gemcitabine resistance by competing with UBR4 for binding to the N-terminal domain of EZH2

**Fig. 3:**
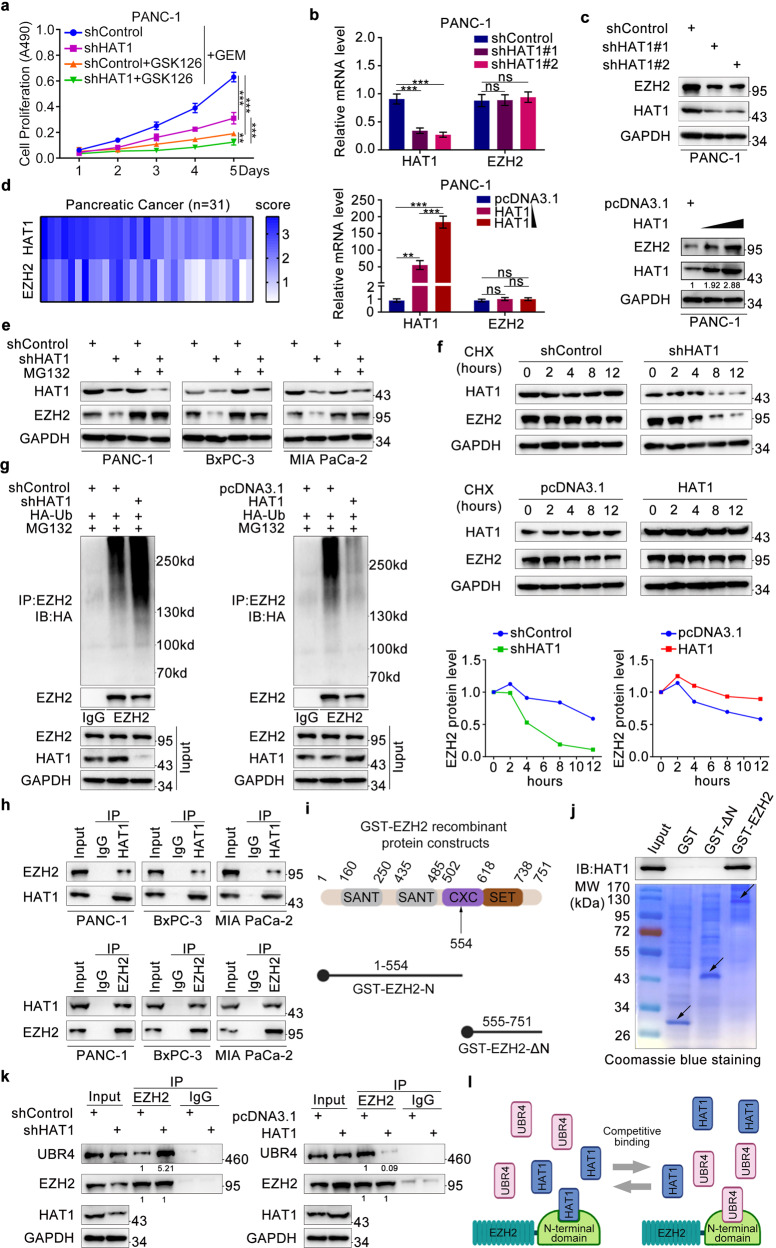
HAT1 stabilizes EZH2 to promote gemcitabine resistance by competing with UBR4 for binding to the N-terminal domain of EZH2. **a** PANC-1 cells were infected with HAT1-specific shRNAs. After infecting 48 h, cells were harvested and treated with GSK126 (10 μM) and gemcitabine for 5 days to perform MTS assay. All data were shown as mean values ± SD (*n* = 3). **P < 0.01; ***P < 0.001. **b, c** PANC-1 cells were infected with lentivirus expressing control, HAT1-specific shRNAs and HAT1-overexpressing plasmid. After infecting 48 h, cells were harvested for RT-qPCR analysis (**b**) and 72 h for western blotting analysis (**c**). Image J software was used to assess relative protein expression level. All data were shown as mean values ± SD (*n* = 3). ns not significant; **P < 0.01; ***P < 0.001. **d** HAT1 and EZH2 IHC score of tissue microarray of pancreatic cancer was plotted the heatmap. **e** PANC-1, BxPC-3, and MIA-PaCa-2 cells were infected with lentivirus expressing control or HAT1-specific shRNAs. After infecting 48 h, cells were treated with MG132 (10 μM) for another 8 h and collected for western blotting analysis. **f** PANC-1 cells were infected with indicated plasmids. After 72 h infection, cells were treated with Cycloheximide (CHX) and then collected for western blotting analysis at different timepoints. **g** PANC-1 were infected with lentivirus expressing control, HAT1-specific shRNAs, pcDNA3.1, or HAT1 plasmid. After 48 h, PANC-1 cells were infected again with HA-Ub plasmid for 24 h and treated with MG132 (10 μM) for another 8 h before harvesting cells. Western blotting analysis was conducted after co-immunoprecipitation. **h** Western blot analysis of endogenous HAT1 and EZH2 proteins reciprocally immunoprecipitated by anti-HAT1 and anti-EZH2 in PANC-1, BxPC-3, MIA-PaCa-2 cells. Immunoblots (IB) were representative of results from three independent experiments (*n* = 3). **i** Two GST-EZH2 recombinant proteins were constructed according to the structural representation: a whole-length GST-EZH2 and an N-terminus-lacking GST-EZH2 (GST-ΔN). **j** Western blot analysis of HAT1 proteins in PANC-1 pulled down by GST or GST-EZH2 recombinant proteins. Immunoblots were representative of results from three independent experiments (*n* = 3). The bottom panel shows Coomassie Blue staining of GST and GST-EZH2 recombinant protein input. Arrows, expected molecular weight. **k** The whole-cell lysate and Co-IP samples were analyzed by western blotting in PANC-1 cells after 48 h being infected with lentivirus expressing control shRNA or HAT1-specific shRNAs. UBR4 coimmunoprecipitated by EZH2 were quantified by Image J software and normalized to the quantified value of immunoprecipitated EZH2. The normalized values were further normalized to the value in cells infected with shControl. IB were representative of results from there independent experiments (*n* = 3). **l** The schematic diagram of HAT1 regulating EZH2.

Several researches showed that EZH2 was a drug-related resistant gene [14, 20, 21], our research also confirmed EZH2 made pancreatic cancer cells become insensitive to gemcitabine (Fig. S4a, b). PVT1 had also been shown to bind EZH2 [22, 23], it was consistent in pancreatic cancer cells. PVT1 did not change the mRNA level of EZH2 (Fig. S4c), but could bind to EZH2 protein (Fig. S4d, e). Thus, we tried to detect the correlation between HAT1 and EZH2. First, the appropriate working concentration and duration of GSK126 (10 μM and 3 days) were screened in pancreatic cancer cells (Fig. S4f). We found that EHZ2 inhibition with GSK126 or silence suppressed HAT1-mediated gemcitabine resistance (Fig. 3a and Fig. S4g-i), suggesting that EZH2 is required for the ability of HAT1 to promote gemcitabine resistance. We also found that the protein but not the mRNA levels of EZH2 were decreased after HAT1 silencing in pancreatic cancer cells. Conversely, forced HAT1 expression increased the protein level of EZH2, although EZH2 mRNA levels remained unchanged (Fig. 3b, c and Fig. S5a, b). Tissue microarray of pancreatic cancer (*n* = 31) was used to conduct IHC analysis and ascertain the relationship between HAT1 and EZH2 in pancreatic cancer (Fig. S5c). The IHC score of HAT1 and EZH2 was calculated and summarized in a heatmap (Fig. 3d). We observed a positive correlation between the protein levels of HAT1 and EZH2 in pancreatic cancer tissues (Spearman correlation coefficient r = 0.5899, *P* = 0.0005; Fig. S5d).

Since HAT1 primarily regulated EZH2 expression at the post-transcriptional level, we hypothesized that HAT1 may regulate EZH2 protein stability. Although EZH2 protein levels were reduced after HAT1 silencing, treatment with the proteasome inhibitor MG132 restored EZH2 protein levels in HAT1-knockdown pancreatic cancer cells (Fig. 3e). Additionally, the half-life of EZH2 protein was significantly shorter in pancreatic cells with HAT1 knockdown than in control cells; HAT1 overexpression extended the half-life of EZH2 protein (Fig. 3f). Further, HAT1 silencing increased the polyubiquitination levels of EZH2, and HAT1 overexpression decreased EZH2 polyubiquitination (Fig. 3g). Co-immunoprecipitation assays revealed an interaction between HAT1 and EZH2 in pancreatic cancer cell lines, regardless of endogenous or exogenous expression (Fig. 3h and Fig. S5e). To identify the EZH2-binding domain of HAT1, we constructed two GST-EZH2 recombinant proteins: a whole-length GST-EZH2 and an N-terminus-lacking GST-EZH2 (GST-ΔN) (Fig. 3i). GST pull-down revealed that EZH2 lacking the N-terminal domain failed to interact with HAT1, indicating that the N-terminal domain of EZH2 is required for its binding to HAT1 (Fig. 3j). The N-terminal WD repeat domain of EZH2 is believed to be a ubiquitin ligase-binding motif [24, 25]; as an E3 ubiquitin ligase, UBR4 may bind to the N-terminus of EZH2 to promote EZH2 polyubiquitination [26]. Thus, we hypothesized that HAT1 binds to the N-terminal domain of EZH2, competing with UBR4 binding. Supporting this hypothesis, HAT1 silencing increased the ability of UBR4 to bind EZH2, whereas HAT1 overexpression had the opposite effect (Fig. 3k). Moreover, restoring HAT1 expression into HAT1-deficient cells led to the decreased interaction between EZH2 and UBR4 (Fig. S5f). These data suggest that HAT1 competes with UBR4 for binding to the N-terminal domain of EZH2, thereby stabilizing EZH2 (Fig. 3l).

### HAT1 stabilizes EZH2 by competing with UBR4 for binding to EZH2

**Fig. 4:**
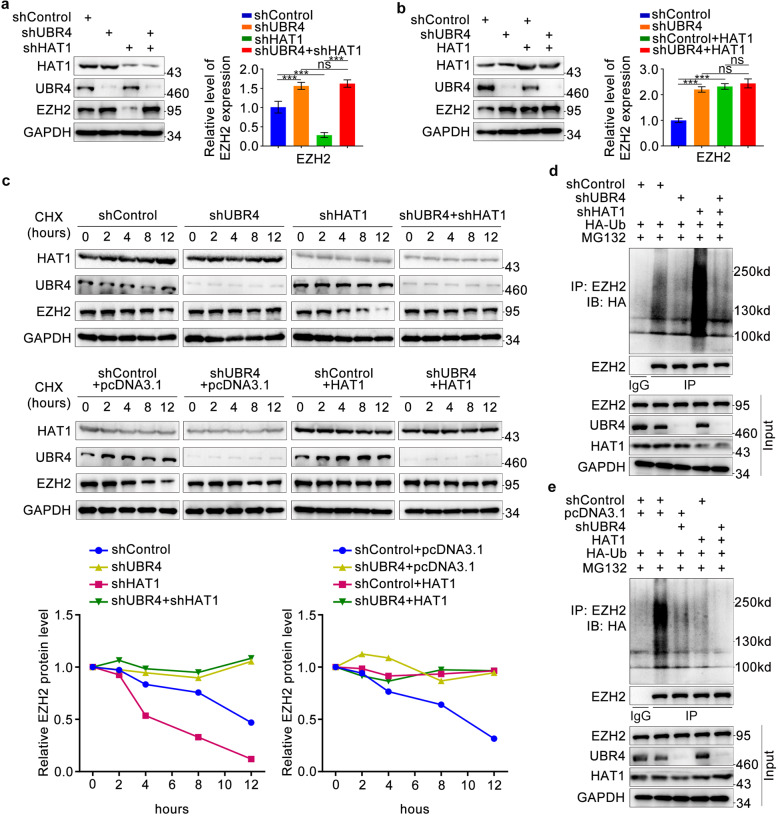
HAT1 stabilizes EZH2 by competing with UBR4 for binding to EZH2. **a,b** PANC-1 cells were infected with lentivirus expressing control, HAT1-specific shRNAs, UBR4-specific shRNAs, and HAT1-overexpressing plasmid. After infecting 72 h, cells were harvested for western blotting analysis (*n* = 3). **c** PANC-1 cells were infected with indicated plasmids. After 72 h infection, cells were treated with Cycloheximide (CHX) and then collected for western blotting analysis at different timepoints. **d**, **e** PANC-1 cells were infected with indicated plasmids. After 48 h, PANC-1 cells were infected again with HA-Ub plasmid for 24 h and treated with MG132 (10 μM) for another 8 h before harvesting cells. Western Blotting analysis was conducted after co-immunoprecipitation.

We also found that UBR4 silencing profoundly increased the protein levels of EZH2 (Fig. 4a, b), as well as extended its half-life (Fig. 4c) and decreased its polyubiquitination levels (Fig. 4d, e), regardless of HAT1 silencing or overexpression. These data further confirmed that HAT1 stabilizes EZH2 by interfering with the binding of UBR4 to the N-terminus of EZH2.

### Preparation and characterization of chitosan (CS)-tripolyphosphate (TPP)-siHAT1

**Fig. 5:**
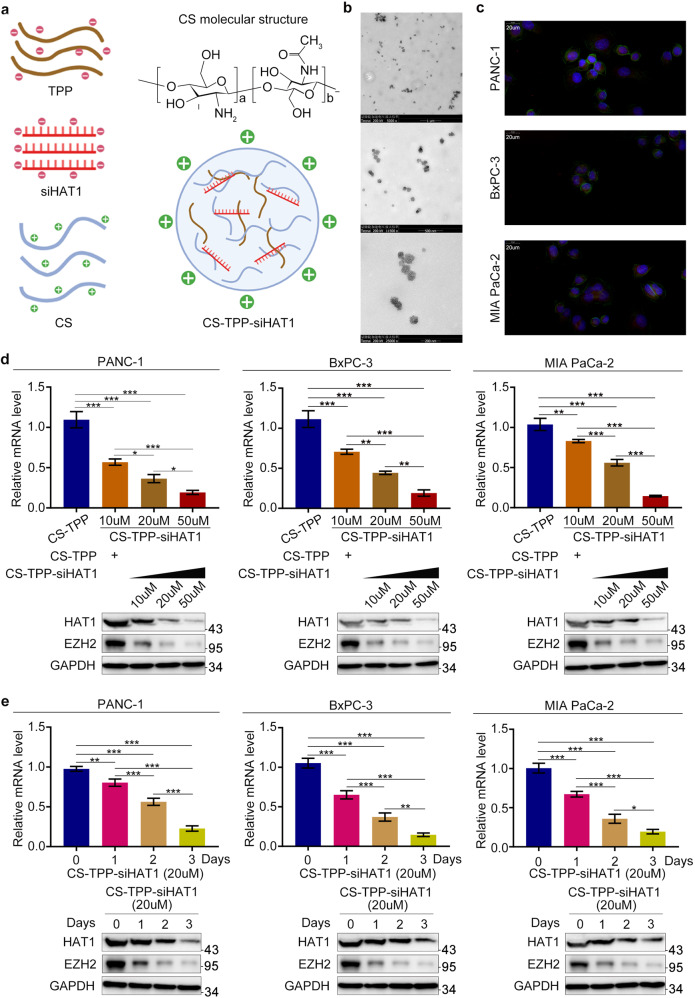
Preparation and characterization of chitosan (CS)-tripolyphosphate (TPP)-siHAT1. **a** The scheme for CS-TPP-siHAT1 preparation. 250 ug siHAT1 was added into 1 mg tripolyphosphate (TPP) solution. Then, the above mixture solution was added into 5 mg chitosan (CS) solution dropwise and stirring slowly for 30 min to prepare the CS-TPP-siHAT1.TPP tripolyphosphate, CS chitosan. **b** Different magnifications TEM images of CS-TPP-siHAT1. **c** The cellular uptake confocal images of CS-TPP-siRNA in PANC-1, BxPC-3, and MIA-PaCa cells. **d** PANC-1, BxPC-3, and MIA-PaCa cells were treated with different CS-TPP-siHAT1 concentration (10 μM, 20 μM, 50 μM) for 3 days. The cells were harvested to extract total protein and RNA respectively, and then RT-qPCR and western blotting were performed to analyzed HAT1, PVT1, and EZH2 expression level. All data were shown as mean values ± SD (*n* = 3). *P < 0.05; **P < 0.01; ***P < 0.001. **e** PANC-1, BxPC-3, and MIA-PaCa cells were treated with 20 μM CS-TPP-siHAT1 for different time (0 days, 1 days, 2 days, 3 days). The cells were harvested to extract total protein and RNA, respectively, and then RT-qPCR and western blotting were performed to analyzed HAT1, PVT1, and EZH2 expression level. All data were shown as mean values ± SD (*n* = 3). *P < 0.05; **P < 0.01; ***P < 0.001.

CS-TPP-siRNA nanoparticles were prepared using an ionic gelation method and through crosslinking the negatively charged phosphate groups of TPP with the positively charged amino groups of CS [27]. At a 5:1 ratio, CS-TPP formed small, positively charged nanoparticles [28]. Hence, we prepared CS-TPP-siRNA nanoparticles by adding 250 μg of siHAT1 to a solution containing 1 mg TPP. This mixture was added dropwise to a solution containing 5 mg CS, yielding CS-TPP-siHAT1 nanoparticles (Fig. 5a). Transmission electron microscopy indicated that CS-TPP-siHAT1 particles had a spherical structure (Fig. 5b). The cellular uptake of CS-TPP-siRNA was also investigated by laser scanning confocal microscopy; strong fluorescent signals were observed in the cytoplasm (Fig. 5c). To assess the ability of CS-TPP-siHAT1 to suppress HAT1 expression, we incubated pancreatic cancer cells with different concentrations of CS-TPP-siHAT1 and for different times. A high concentration of CS-TPP-siHAT1 and prolonged incubation provided superior HAT1 downregulation, which also had a significant inhibitory effect on EZH2 and PVT1 (Fig. 5d, e and Fig. S5g).

### CS-TPP-siHAT1 augments the ability of gemcitabine to inhibit pancreatic cancer cell growth

**Fig. 6:**
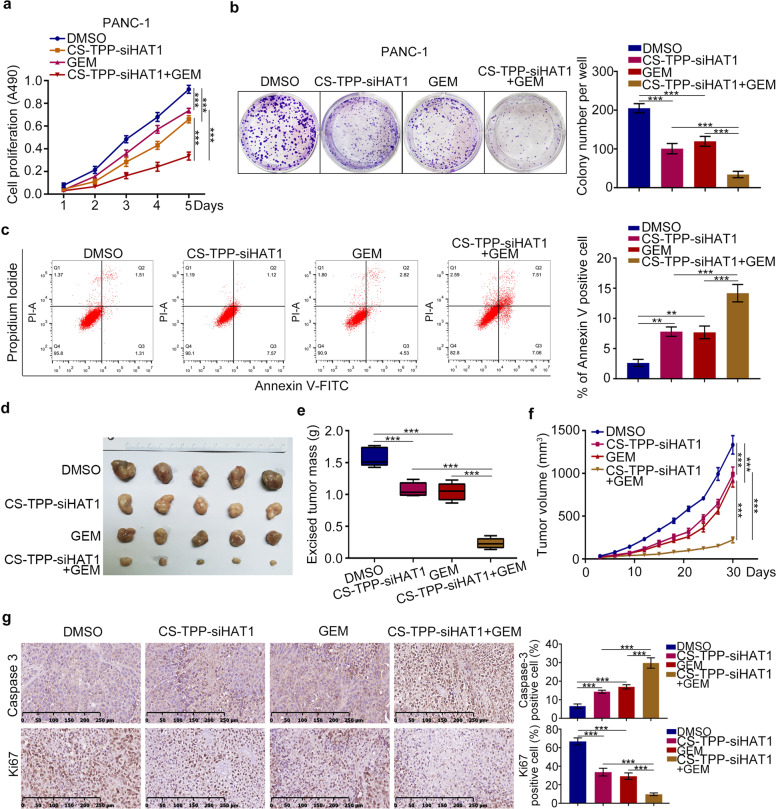
CS-TPP-siHAT1 augments the ability of gemcitabine to inhibit pancreatic cancer cell growth. **a–c** PANC-1 cells were treated with CS-TPP-siHAT1 alone, gemcitabine alone or the combination for MTS assay (**a**), clone formation assay (**b**), and Annexin-V/Propidium Iodide assay (**c**). All data were shown as mean values ± SD (*n* = 3). **P < 0.01; ***P < 0.001. **d–f** PANC-1 cells were subcutanousely injected into the nude mice. These mice were injected intraperitoneally with CS-TPP-siHAT1 (50 μM) alone, gemcitabine (10 mg/kg) alone or the combination every 3 days. The tumor volume was measured every 3 days and all tumors were harvested for photograph and weight after 30 days (*n* = 5). ***P < 0.001. **g** All tumors were conducted caspase-3 and Ki67 analysis by IHC staining. All data were shown as means values ± SD (*n* = 5). ***P < 0.001.

Next, we assessed the ability of CS-TPP-siHAT1 to enhance the cytotoxic effects of gemcitabine and found that the combination of CS-TPP-siHAT1 with gemcitabine was more potent in inhibiting cell proliferation than CS-TPP-siHAT1 or gemcitabine alone (Fig. 6a, b and Fig. S6a, b). Additionally, Annexin-V/PI staining revealed that CS-TPP-siHAT1 augmented the proapoptotic effects of gemcitabine (Fig. 6c). In nude mice, CS-TPP-siHAT1 and gemcitabine were used to treat the nude, the detailed information was as follows (Fig. S6c). The combination of CS-TPP-siHAT1 with gemcitabine exhibited the most potent tumor-suppressive effects among all groups (Fig. 6d–f). The delaminated tumors were also extracted proteins to analyze the expression of HAT1 (Fig. S6d). Additionally, tumors from mice treated with CS-TPP-siHAT1 combined with gemcitabine showed the highest caspase-3 levels and lowest Ki67 levels (Fig. 6g). Moreover, GR-PANC-1 was also used to further attest the synergistic effect, which showed that gemcitabine suppressed the growth of GR-PANC-1 and promoted the apoptosis of GR-PANC-1 after inhibiting HAT1 with CS-TPP-siHAT1 (Fig. S6e-g). These data suggested CS-TPP-siHAT1 and gemcitabine have a synergistic antitumor effect in pancreatic cancer.

## Discussion

Despite recent progress in cancer therapeutics, the prognosis of pancreatic cancer remains poor, primarily due to the development of gemcitabine resistance. The mechanisms underlying resistance to gemcitabine remain unclear and involve alterations in drug transporters, proteases, transcription factors, and drug metabolism enzymes [29, 30, 7]. These alterations can be internal to pancreatic cancer cells or induced by components of the tumor microenvironment [31].

HAT1 was first identified as a classical B type histone acetyltransferase mediating the acetylation of histone H4 N-terminus [32]. As an epigenetic modifier, acetyl moieties can be found on lysine residues of cellular proteins (e.g., histones, transcription factors, nuclear receptors, and enzymes). In addition to regulating gene expression, protein acetylation plays a critical role in replication-dependent chromatin assembly and DNA damage repair [33, 34]. HAT1 mutations are frequent in tumors, leading to abnormal gene expression and promoting resistance to chemotherapeutic agents. For example, HAT1 was found to promote liver cancer cell proliferation and induce cisplatin resistance [17]. In melanoma, HAT1 was shown to catalyze histone H4 acetylation, thereby driving resistance to BET inhibitors [18]. In addition to HAT1 mutations, HAT1 overexpression is also frequent in multiple cancer types, including colon cancer [35], esophageal cancer [36], and lymphoma [37], exacerbating tumor malignancy. Hence, HAT1-mediated resistance to chemotherapy is an internal characteristic of tumor cells. We have previously shown that HAT1 was overexpressed in pancreatic cancer and that HAT1 silencing reduced the expression of PD-L1 on the surface of pancreatic cancer cells in a BRD4-dependent manner, improve the therapeutic efficacy of immune checkpoint blockade [38]. These findings suggest that HAT1 may represent an important therapeutic target in pancreatic cancer.

In this study, we found that HAT1 overexpression in pancreatic cancer cells promoted gemcitabine resistance and that HAT1 silencing restored sensitivity to the antitumor effects of gemcitabine. To acquire further insight into the molecular mechanism underlying HAT1-mediated gemcitabine resistance, we knocked silenced HAT1 expression in PANC-1 cells and performed RNA sequencing, which indicated PVT1 as a potential HAT1 target gene. PVT1 is a lncRNA located on the human chromosome 8q24 [39, 40] near the oncogene MYC gene, the expression of which is enhanced by PVT1 [14, 40]. Mounting evidence suggests that PVT1 has oncogenic functions in various tumors. Notably, PVT1 enhanced Bcl2 expression in gastric cancer cells, thereby inhibiting apoptosis and promoting resistance to 5-fluorouracil [41]. Additionally, PVT1 was found to upregulate the expression of numerous drug resistance-related molecules (e.g., MDR1 and MRP1) and inhibit apoptosis signaling, promoting cisplatin resistance in colorectal cancer. Notably, a previous genome-wide screening identified PVT1 as a critical regulator of gemcitabine sensitivity in pancreatic cancer [13]. Here, we confirmed the role of HAT1 in regulating PVT1 expression and identified BRD4 as a key player of the HAT1-mediated regulation of PVT1 expression.

Previous studies have shown that by forming a complex with EZH2, PVT1 promoted cell proliferation and inhibited apoptosis in liver cancer and thyroid cancer cells [23, 42]. The histone methyltransferase EZH2 suppresses gene expression by catalyzing the trimethylation of histone H3 lysine 27 (H3K27me3) [43, 44]. EZH2 has also been implicated in multidrug resistance in gastric cancer and ovarian cancer [45, 46]. Importantly, EZH2 has been shown to promote drug resistance by suppressing p27^Kip1^ expression in pancreatic cancer cells [12]. Our study was consistent with previous research results, which showed EZH2 regulated the gemcitabine sensitivity and PVT1 could bind to EZH2. More importantly, we found that HAT1 enhanced the expression of EZH2 in pancreatic cancer cells in this study. We also found that HAT1 regulated EZH2 expression at the post-transcriptional rather than transcriptional level. Moreover, we found that HAT1 interacted with EZH2, increasing the stability of the latter. Jalan-Sakrikar N et al. found that the E3 ubiquitin ligase UBR4 could bind to the N-terminus of EZH2, promoting EZH2 ubiquitination and subsequent degradation [26]. Consistent with these results, we found that HAT1 could bind to the N-terminus of EZH2, interfering with the ability of UBR4 to interact with EZH2 (Fig. 3l).

This study suggests that HAT1 regulates the sensitivity of pancreatic cancer to gemcitabine by regulating PVT1/EZH2 complex, highlighting the importance of HAT1 in the development of gemcitabine resistance in pancreatic cancer. Although we provide strong evidence that HAT1 inhibition may suppress tumor growth, reverse drug resistance, and improve the prognosis of pancreatic cancer, there are currently no HAT1 small molecule inhibitors. High-throughput screening approaches have led to the identification of potential drug candidates; however, most of these compounds exhibited moderate efficacy and specificity [47]. Therefore, future studies are urgently needed to develop specific and potent HAT1 inhibitors. Nanoparticles carrying siRNA have emerged as promising alternatives of small molecule inhibitors [48–50]. Here, we used a CS-TPP carrier to deliver siHAT1 and suppress HAT1 expression in pancreatic cancer cells. CS-TPP-siHAT1 nanoparticles have proved effective in inhibiting HAT1 expression and augmenting the antitumor effects of gemcitabine in pancreatic cancer. The effectiveness and safety of CS-TPP-siHAT1 nanoparticles required further investigation in a clinical setting.

## Conclusions

Our data strongly support that HAT1 upregulates PVT1 and promotes gemcitabine resistance in pancreatic cancer by enhancing BRD4 binding to the PVT1 promoter. We also show that HAT1 prevents EZH2 degradation by preventing UBR4 binding to the N-terminal domain of EZH2. Our findings suggest that HAT1-induced gemcitabine resistance in pancreatic cancer may be mediated by the PVT1/EZH2 complex. Finally, we show that CS-PTT-siHAT1 nanoparticles suppress HAT1 expression and augment the antitumor effects of gemcitabine in pancreatic cancer cells. Collectively, the findings presented here suggest that HAT1 may be a valuable therapeutic target in pancreatic cancer.

## Materials and methods

### Cell lines and cell culture

Pancreatic cancer cell lines (PANC-1, BxPC-3, and MIA-PaCa-2) were purchased from the Chinese Academy of Science Cell Bank. Cell lines were cultured in RPMI 1640 medium (Gibco, USA) containing 10% fetal bovine serum (FBS; Gibco, USA), 100 IU/mL penicillin, and 100 μg/mL streptomycin (Gibco, USA). Plasmocin (InvivoGen) was routinely added to the medium to eliminate mycoplasma. All cell lines were maintained at 37 °C in a 5% CO_2_ incubator.

### Cell transfection

Cells were transfected with different plasmids using Lipofectamine 2000 Reagent (Thermo Fisher Scientific). Small hairpin RNAs (shRNAs) targeting HAT1 (shHAT1, shBRD4, shUBR4, shPVT1, and shEZH2) and overexpression plasmid (pcDNA3.1 backbone vector, HAT1, BRD4, and PVT1) were obtained from Shanghai GeneChem Co., Ltd. Opti-MEM medium (Gibco, USA) was used to prepare the transfection mixtures. Six hours after transfection, the Opti-MEM medium was replaced with 10% FBS-containing RPMI 1640 medium. The sequences of shHAT1 and shBRD4 are provided in Table 1.

**Table 1:** The sequences of gene-specific shRNAs.

shHAT1#1	5′- CCGGGCTACATGACAGTCTATAATTCTCGAGAATTATAGACTGTCATGTAGCTTTTTG-3′
shHAT1#2	5′- CCGGCCGTGTTGAATATGCATCTAACTCGAGTTAGATGCATATTCAACACGGTTTTTG-3′
shHAT1#3	5′-CCGGGCAAGGATTCAATGAAGATATCTCGAGATATCTTCATTGAATCCTTGCTTTTTG-3′
shBRD4#1	5′-CCGGCCTGGAGATGACATAGTCTTACTCGAGTAAGACTATGTCATCTCCAGGTTTTTG-3′
shBRD4#2	5′-CCGGCAGTGACAGTTCGACTGATGACTCGAGTCATCAGTCGAACTGTCACTGTTTTTG-3′
shUBR4#1	5′-CCGGGCCGCTGATGAGGGATATAAACTCGAGTTTATATCCCTCATCAGCGGCTTTTTTG-3′
shUBR4#2	5′-CCGGCCACCATCAAAGACTTACATTCTCGAGAATGTAAGTCTTTGATGGTGGTTTTTTG-3′
shPVT1#1	5′-ACCGGACTTGAGAACTGTCCTTACCGAAGTAAGGACAGTTCTCAAGTCC-3′
shPVT1#2	5′-CACCGCTTCTCCTGTTGCTGCTAGTCGAAACTAGCAGCAACAGGAGAAGC-3′
shEZH2#1	5′-CCGGCCCAACATAGATGGACCAAATCTCGAGATTTGGTCCATCTATGTTGGGTTTTTG-3′
shEZH2#2	5′-CCGGTATGATGGTTAACGGTGATCACTCGAGTGATCACCGTTAACCATCATATTTTTG-3′

### Lentiviral and the construction of stable cell lines

Lentiviral particles carrying gene-specific shRNAs and negative control shRNA (hU6-MCS-CBh-gcGFP-IRES-puromycin) were used to infect pancreatic cancer cells. Briefly, pancreatic cancer cells were seeded in six-well plates (2 × 10^5^ cells per well). The day after, 1 mL of DMEM medium containing the viral particles and 40 μL of Hitrans G reagent was added to each well. After 16 h, the viral solution was replaced with complete growth medium. Puromycin was used to select infected cells.

### Antibodies and chemicals

In this study, the following antibodies were used: anti-HAT1 (11432-1-AP, Proteintech; 1:1000 dilution), anti-EZH2 (21800-1-AP, Proteitech; 1:1000 dilution), anti-UBR4 (ab86738, Abcam; 1:2000 dilution), anti-caspase-3 (19677-1-AP, Proteintech; 1:2000 dilution), anti-GAPDH (10494-1-AP, Proteintech; 1:2000 dilution), anti-HA (51064-2-AP, Proteintech; 1:1000 dilution), anti-Flag (20543-1-AP, Proteintech; 1:1000 dilution), anti-H4 (16047-1-AP, Proteintech; 1:1000 dilution), anti-H4ac (AB_2687872, 1:1000 dilution). The following chemicals were used: gemcitabine (T0251, Topscience; 50 nM in vitro and 10 mg/kg in vivo), JQ1 (Cat. No. S7110, Selleck; working concentration 10 μM), MG132 (HY-13259, MedChemExpress; working concentration 10 μM), and GSK126 (HY-13470, MedChemExpress; working concentration 10 μM).

### Immunohistochemistry (IHC)

Pancreatic cancer tissue microarrays (HPan-Ade060CD-01) were purchased from Shanghai Outdo Biotech Co., Ltd and obtained the ethical committee approval. Tissues were stained with anti-HAT1 (1:2000 dilution) and anti-EZH2 (1:1000 dilution). The IHC score was determined independently by two experienced pathologists blinded to the study groups. The staining intensity was scored as follows: 1, weak staining at ×100 magnification and little or no staining at ×40 magnification; 2, moderate staining at ×40 magnification; 3, strong staining at ×40 magnification.

### Cell proliferation and colony formation assay

The MTS (3-(4,5-dimethylthiazol-2-yl)-5-(3-carboxymethoxyphenyl)-2-(4-sulfophenyl)-2H-tetrazolium) reagent (ab197010, Abcam, USA) was used to assess cell proliferation in vitro. Cells were seeded in 96-well plates (2000 cells in 100 μl DMEM per well) and cultured for 3 days. Subsequently, 20 μl MTS reagent was added to each well, and cells were incubated for 4 h in the dark. Optical absorbance at 490 nm was measured on a microplate reader after adding 200 μl DMSO.

Cells were seeded in six-well plates (500 cells/well) for colony formation assays. The cell growth medium was replaced every 3 days. After 2 weeks, cells were fixed with 4% paraformaldehyde (30 min) and stained with crystal violet (20 min).

### The preparation of gemcitabine-resistant PANC-1

Gemcitabine was dissolved in dimethyl sulfoxide (DMSO) at a concentration of 1 mM. Then, gemcitabine solution was diluted to a working concentration of 50 nM with RPMI 1640 medium containing 10% FBS. The pancreatic cancer cell line PANC-1 was cultured with the above configured medium for 2 weeks in 5% CO2 at 37 °C. Then the cells were cultured for another 2 weeks without gemcitabine. The above process was repeated three times. The selected cells could resist gemcitabine and the cells were named GR-PANC-1.

### Western blotting

Cells were lysed by sonication in RIPA lysis buffer (P0013B, Beyotime) containing 1% protease inhibitor. The cell lysate was centrifuged at 12,000 rpm and 4 °C, and the supernatant was collected and boiled for 10 min at 95 °C. The protein concentration was measured using Lowry method with protein concentration determination kit (Solarbio, PC0030). Subsequently, proteins were resolved by SDS-PAGE and transferred onto PVDF membranes (Pierce Biotechnology, USA). Membranes were blocked with 5% skim milk for 1 h at room temperature and incubated with primary antibodies overnight at 4 °C. The next day, the membranes were washed three times and incubated with the respective secondary antibody for 1 h at room temperature. Signal was developed using ECL chemiluminescence reagent and X-ray films.

### Quantitative real-time PCR (qRT-PCR)

Total RNA was extracted using TRIzol reagent (15596026, Invitrogen, USA). PrimeScript™ RT kit (Takara, Japan) was used to synthesize cDNA, which was subsequently amplified using TB Green™ Fast qPCR Mix (Takara, Japan). Relative gene expression was calculated using the 2^-ΔCq^ method after normalizing to *GAPDH* levels. The sequences of the qRT-PCR primers are shown in Table 2.

**Table 2:** The sequences of RT-qPCR primers.

Gene	Forward primer (5′-3′)	Reverse primer (5′-3′)
HAT1	GGATGGAGCTACGCTCTTTG	GGATGGATCTTCCGCTGTAA
PVT1	ATAGATCCTGCCCTGTTTGC	CATTTCCTGCTGCCGTTTTC
EZH2	CCCTGACCTCTGTCTTACTTGTGGA	ACGTCAGATGGTGCCAGCAATA
BRD4	ACCTCCAACCCTAACAAGCC	TTTCCATAGTGTCTTGAGCACC
GAPDH	ACCCACTCCTCCACCTTTGAC	TGTTGCTGTAGCCAAATTCGTT

### Co-immunoprecipitation (Co-IP) and chromatin immunoprecipitation (ChIP)

Cells were lysed in western/IP lysis buffer for 30 min at 4 °C, and the cell lysate was centrifuged for 10 min at 12,000 rpm and 4 °C. The supernatant was incubated with protein A/G agarose beads (Thermo Fisher Scientific, USA) overnight at 4 °C with gentle agitation. The protein A/G agarose beads were collected and washed seven times with western/IP lysis buffer. Subsequently, proteins were resolved by SDS-PAGE.

According to the manufacturer’s protocols, chromatin Immunoprecipitation (ChIP) assay was carried out through utilizing the Chromatin Extraction Kit (Abcam, ab117152, USA) and ChIP Kit Magnetic-One Step (Abcam, ab156907, USA) [38, 51]. BRD4 antibody (Cell Signaling Technology, 13440, working concentration 1:50) was used to perform ChIP assay for precipitating the promoter of PVT1. The precipitated DNA fragments were further amplified via quantitative real-time PCR with a PCR kit (Takara Bio Inc., Japan) based on the manufacturer’s instructions [38, 52]. The sequences of the primers used for ChIP-qPCR are provided in Table 3.

**Table 3:** The sequence of ChIP-qPCR primers.

ChIP target	Gene	Forward primer (5′-3′)	Reverse primer (5′-3′)
BRD4	PVT1	AGGGATGCGCTGTGAGTAGT	TTCTGACTGCAGAGGGGTCT

### RNA immunoprecipitation (RIP) assay

According to the manufacturer’s protocols, RNA immunoprecipitation (RIP) assay was performed through using Magna RIP RNA-Binding Protein Immunoprecipitation Kit (Millipore, MA, USA). The supernatant of cell lysate was extracted and then mixed up with treated beads to incubate for 6 h. Then the RIP wash buffer was used to wash the beads for seven times. At last, qRT-PCR analysis was conducted to analyze the purified RNA.

### RNA pull-down assay

mMESSAGE mMACHINE T7 Kit (Ambion, USA) and RNeasy Mini Kit (Qiagen, Valencia, CA) were used to transcribe PVT1 and the antisense RNA in vitro, and Pierce RNA 3′ End Desthiobiotinylation Kit (Thermo Scientific, USA) was utilized to conduct biotin labeling. One milligram total protein extracts were added to 50 pmol of biotin-labeled PVT1 for incubating 1 h, then mixed up with 60 µL of Streptavidin Beads (Invitrogen) for another 1 h. Finally, Western blotting analysis was used to detect the proteins.

### Flow cytometry

Annexin-V-FITC/PI kit (AntGene, China) was used to assess apoptosis. Cells treated with different agents were harvested and washed with phosphate buffer saline (PBS). Cells were incubated with annexin-V-FITC and PI according to the manufacturer’s instructions. Flow cytometry was performed on BD FACSCelesta (BD Biosciences, USA), and the data were analyzed using FlowJo

### Confocal imaging

Pancreatic cancer cells were seeded into slide chambers, and after overnight incubation, cells were incubated with chitosan (CS)-tripolyphosphate (TPP)-siHAT1 (10 μg/mL) for 4 h. Cells were washed with PBS, fixed in 4% paraformaldehyde, and permeabilized with 0.2% Triton X-100 for 5 min. Subsequently, cells were treated with 100 nM FITC-phalloidin for 1 h at 37 °C and in the dark. Cell nuclei were counterstained with Hoechst 33342 for 5 min. Stained cells were observed under a laser confocal microscope.

### Subcutaneous xenotransplantation tumor model

Nude mice (18–20 g, 5 weeks old, male) were purchased from Vitalriver (Beijing, China) and maintained under specific pathogen-free (SPF) conditions. Mice were randomly divided into groups (five mice per group) and subcutaneously injected with 3 × 10^6^ PANC-1 cells. The volume was measured every 3 days (volume = (L × W^2^)/2). The treatment protocols with drugs were as follows: When the tumors reached to 50 mm^3^, experimental groups were injected intraperitoneally gemcitabine (10 mg/kg) or CS-TPP-siHAT1 (50 μM) with every 3 days, control groups were injected intraperitoneally equal DMSO or CS-TPP. After 30 days, mice were sacrificed, and tumors were excised and weighed. All animal experimental procedures were approved by the Ethics Committee of Tongji Medical College, Huazhong University of Science and Technology.

### Glutathione S-transferase (GST) pull-down assay

GST fusion proteins were immobilized on glutathione-sepharose beads (GE Healthcare Life Sciences), and the beads were incubated with cell lysis buffer (20 mmol/L Tris-HCl [pH 7.5], 150 mmol/L NaCl, 0.1% Nonidet P40, 1 mmol/L dithiothreitol [DTT], 10% glycerol, 1 mmol/L EDTA, 2.5 mmol/L MgCl_2_, and 1 μg/mL leupeptin) for 4 h. After incubation and washing, the beads were resuspended in protein loading buffer and subjected to SDS-PAGE.

### Statistical analysis

All experiments were replicated for three times. All data were analyzed using two-tailed with GraphPad Prism 7.0. Student’s *t*-test, one-way analysis of variance (ANOVA), and two-way ANOVA was used to evaluate statistical significance (similar variance). *P* values <0.05 were considered statistically significant. All values were expressed as means ± SD.

## Supplementary information

supplementary figure 1

supplementary figure 2

supplementary figure 3

supplementary figure 4

supplementary figure 5

supplementary figure 6

supplementary information

## Data Availability

All data generated or analyzed during this study are included in this published article and its supplementary information files.
