# Supplementary figures and images for: Histone acetyltransferase 1 promotes gemcitabine resistance by regulating the PVT1/EZH2 complex in pancreatic cancer
Source: Cell Death Dis. 2021 Sep 25;12(10):878. doi: 10.1038/s41419-021-04118-4 (PMC8464605; doi:10.1038/s41419-021-04118-4)

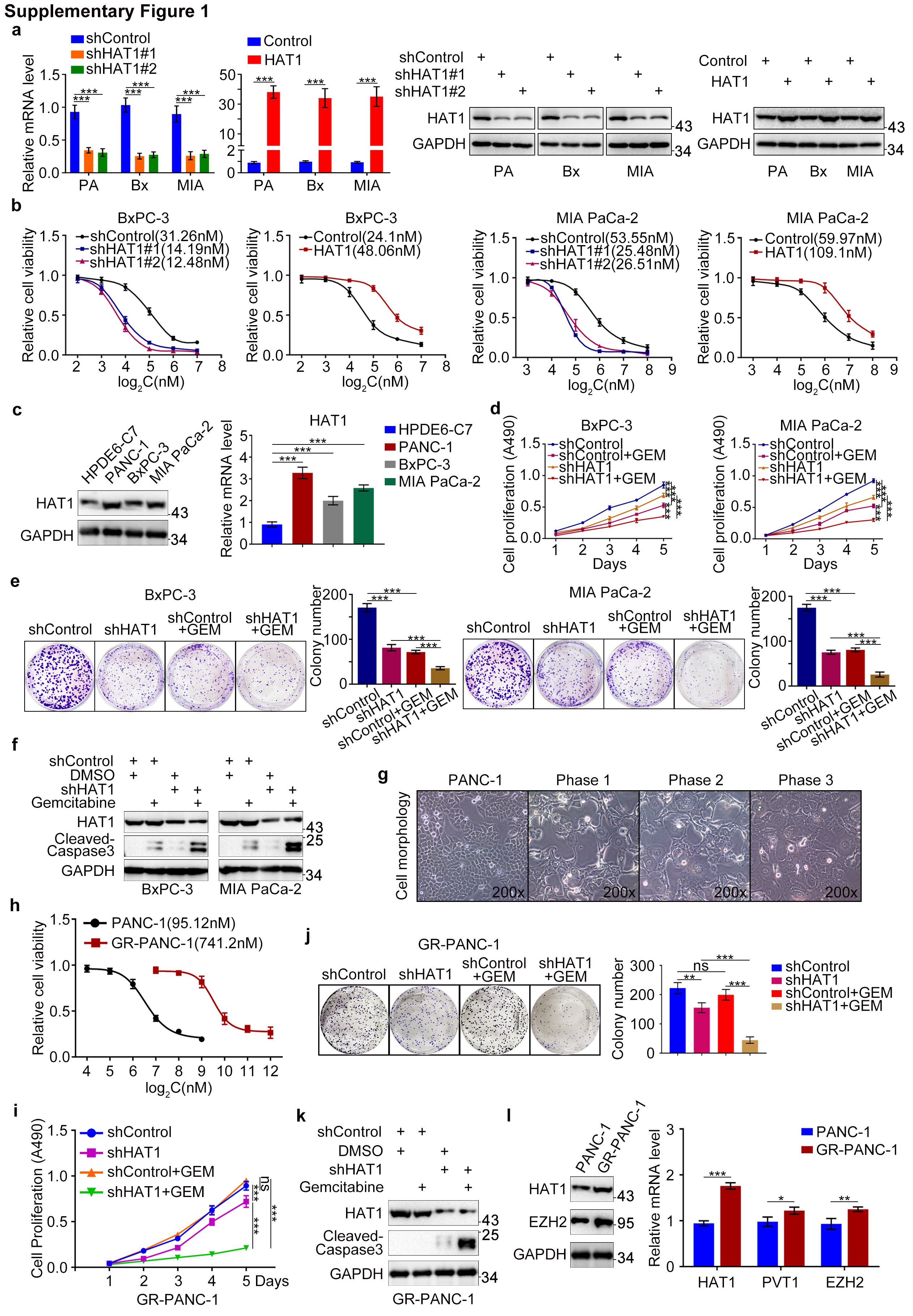

Supplement: Supplementary file 1 — supplementary figure 1 [file 41419_2021_4118_MOESM1_ESM.jpg]

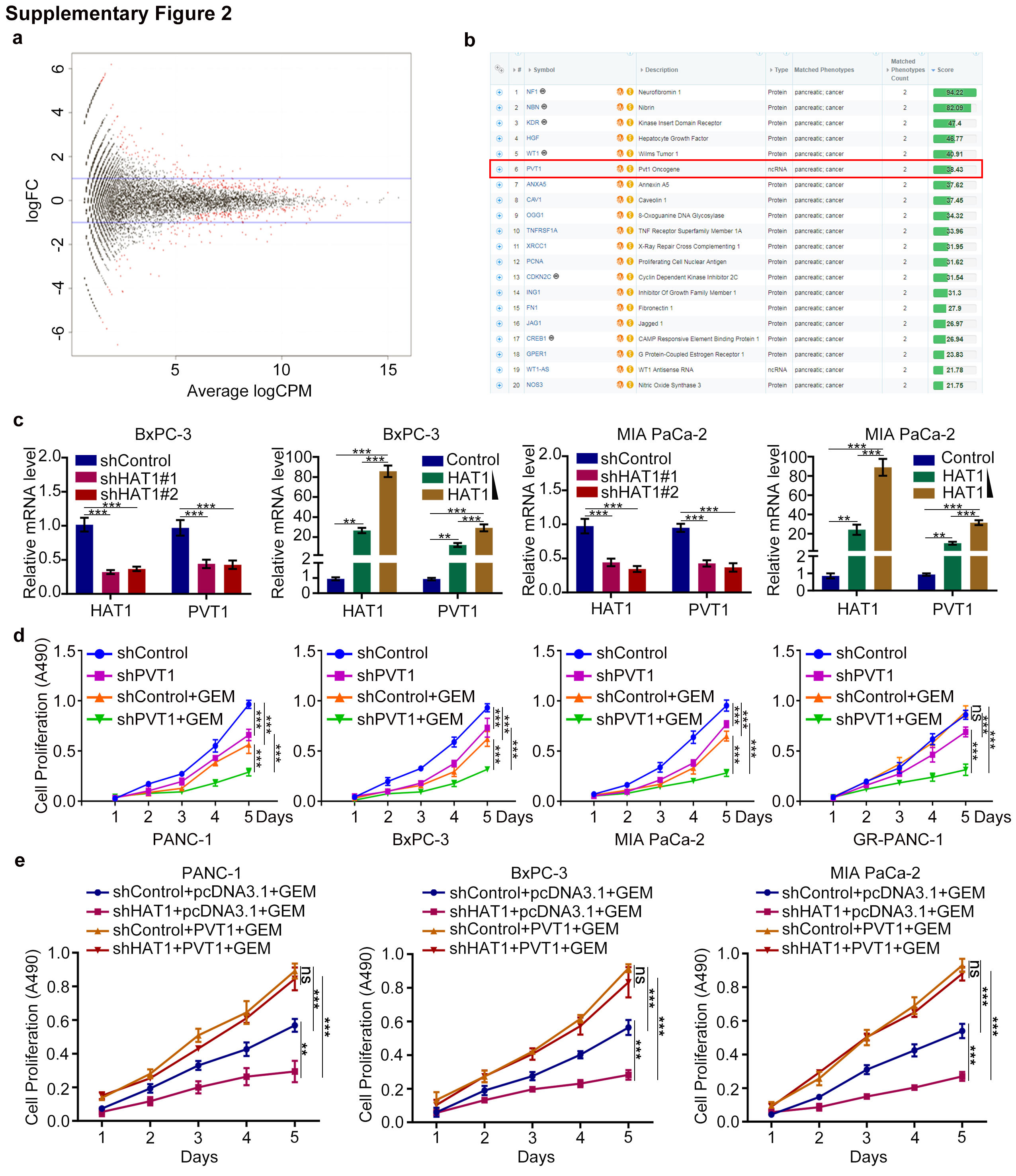

Supplement: Supplementary file 2 — supplementary figure 2 [file 41419_2021_4118_MOESM2_ESM.jpg]

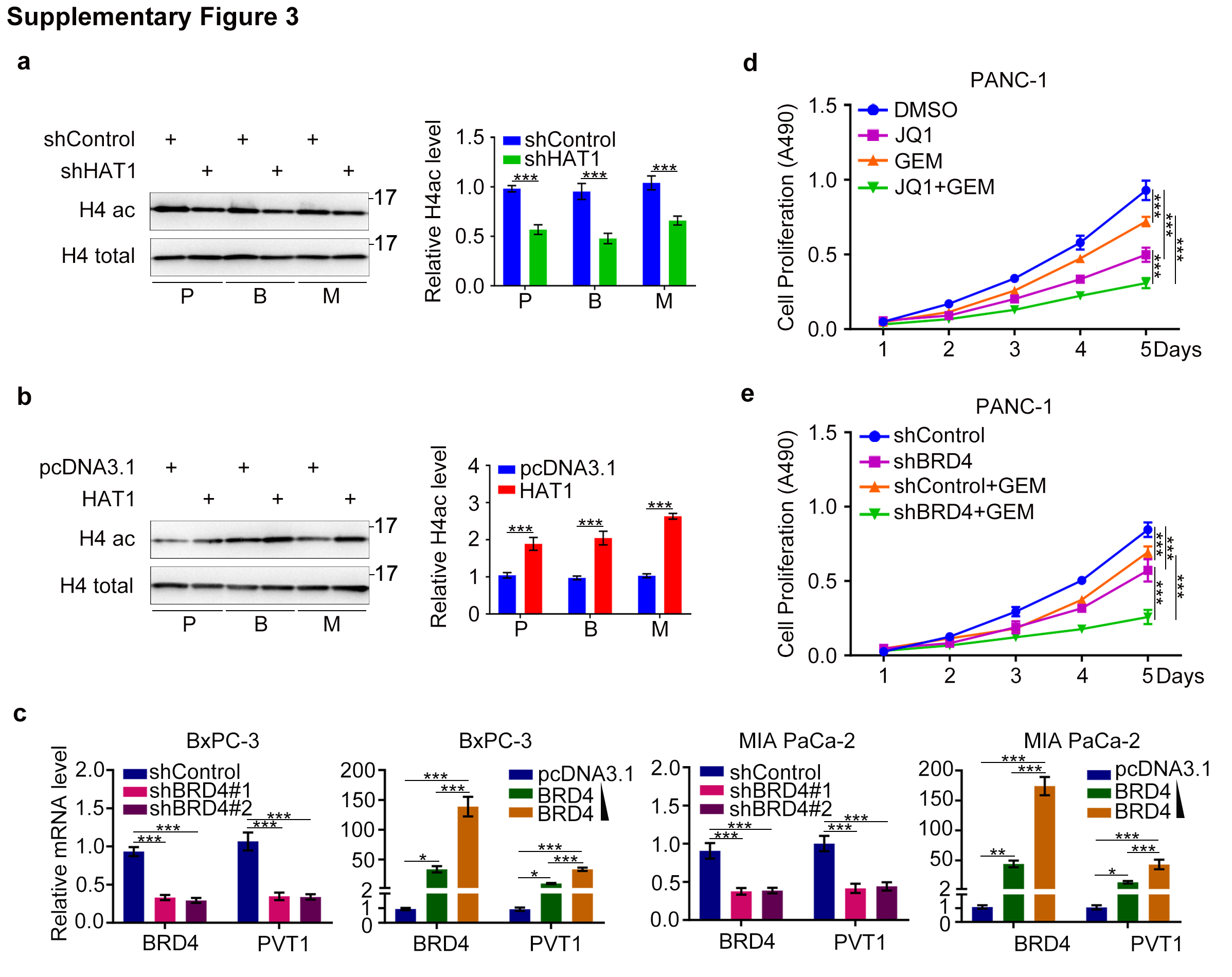

Supplement: Supplementary file 3 — supplementary figure 3 [file 41419_2021_4118_MOESM3_ESM.jpg]

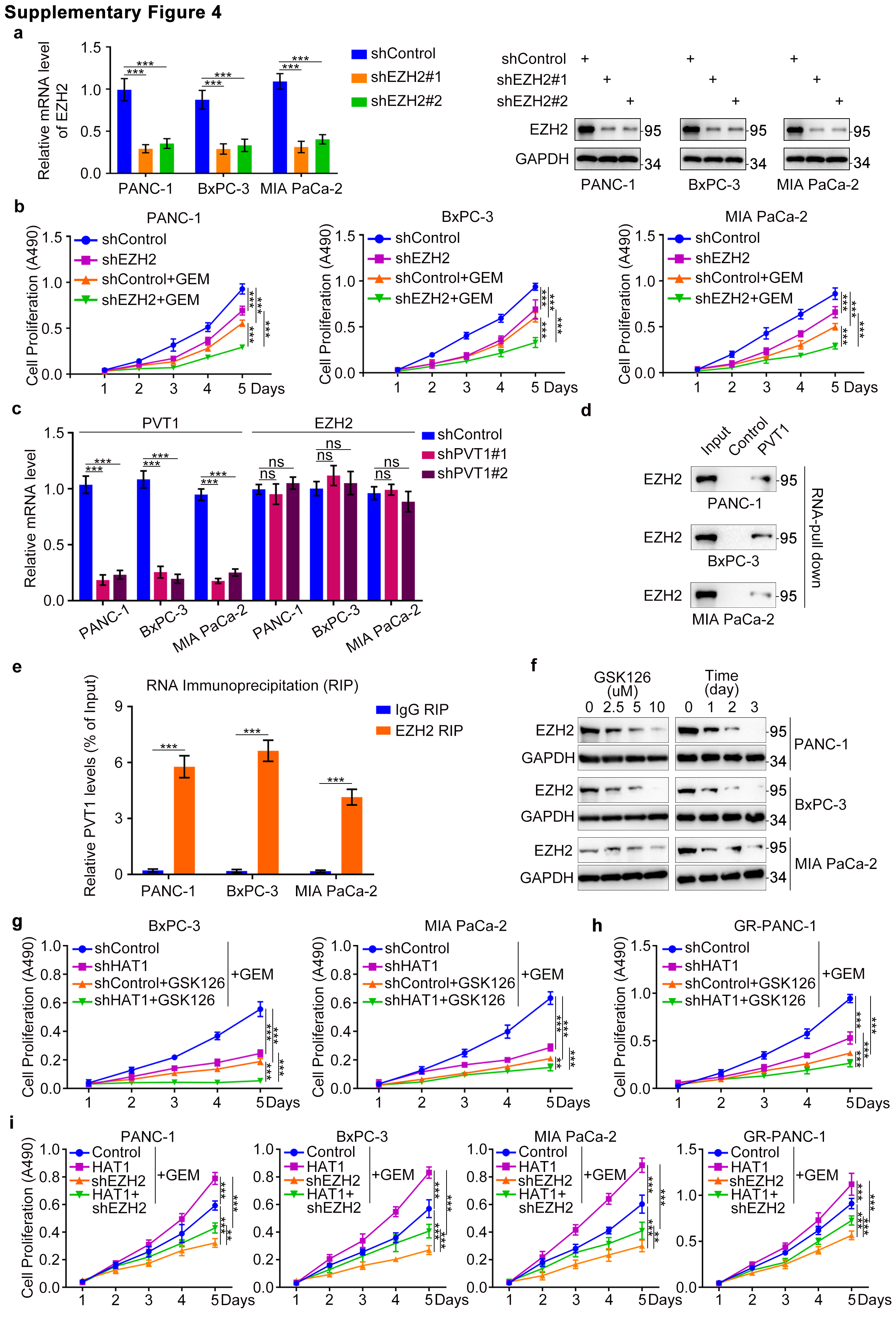

Supplement: Supplementary file 4 — supplementary figure 4 [file 41419_2021_4118_MOESM4_ESM.jpg]

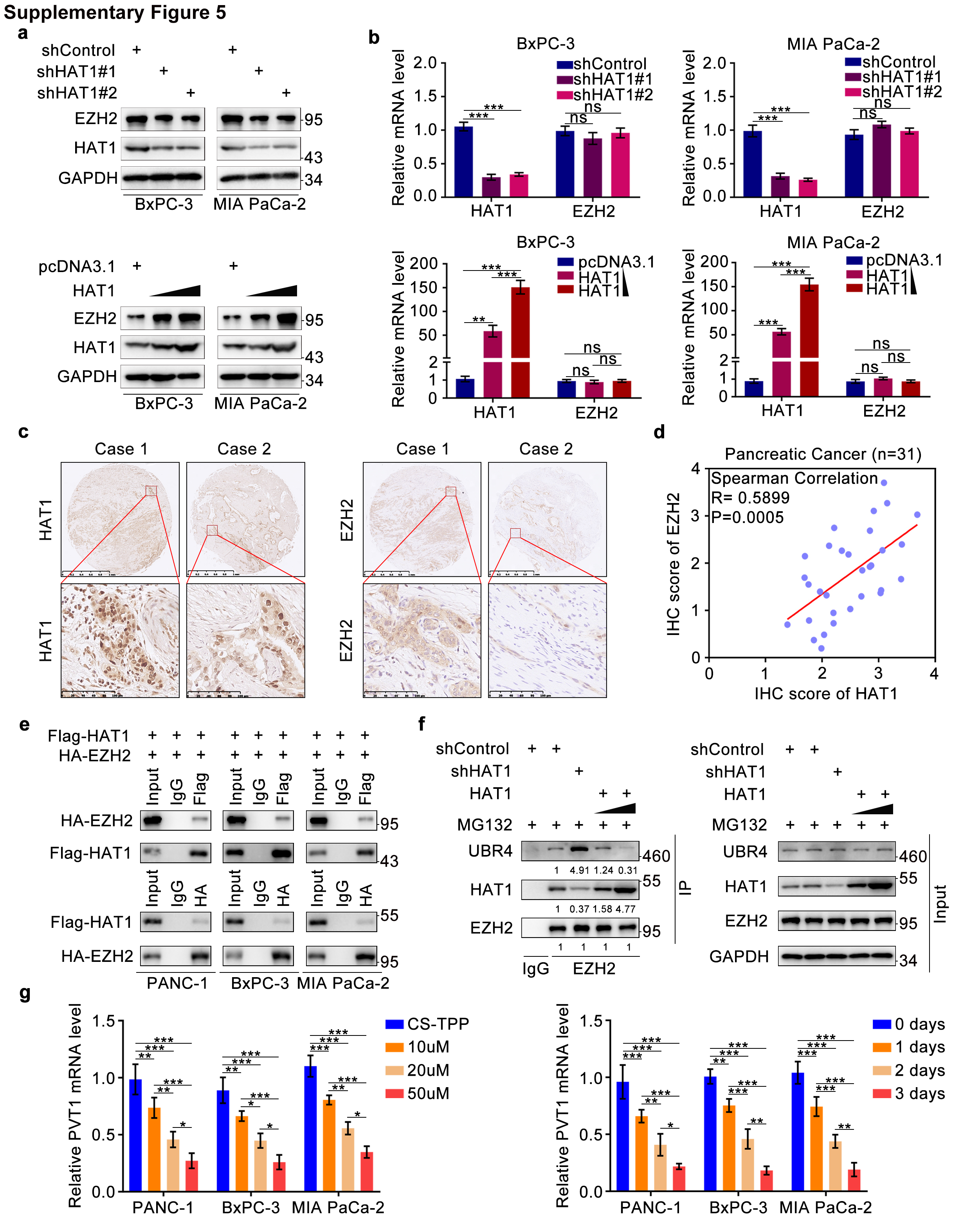

Supplement: Supplementary file 5 — supplementary figure 5 [file 41419_2021_4118_MOESM5_ESM.jpg]

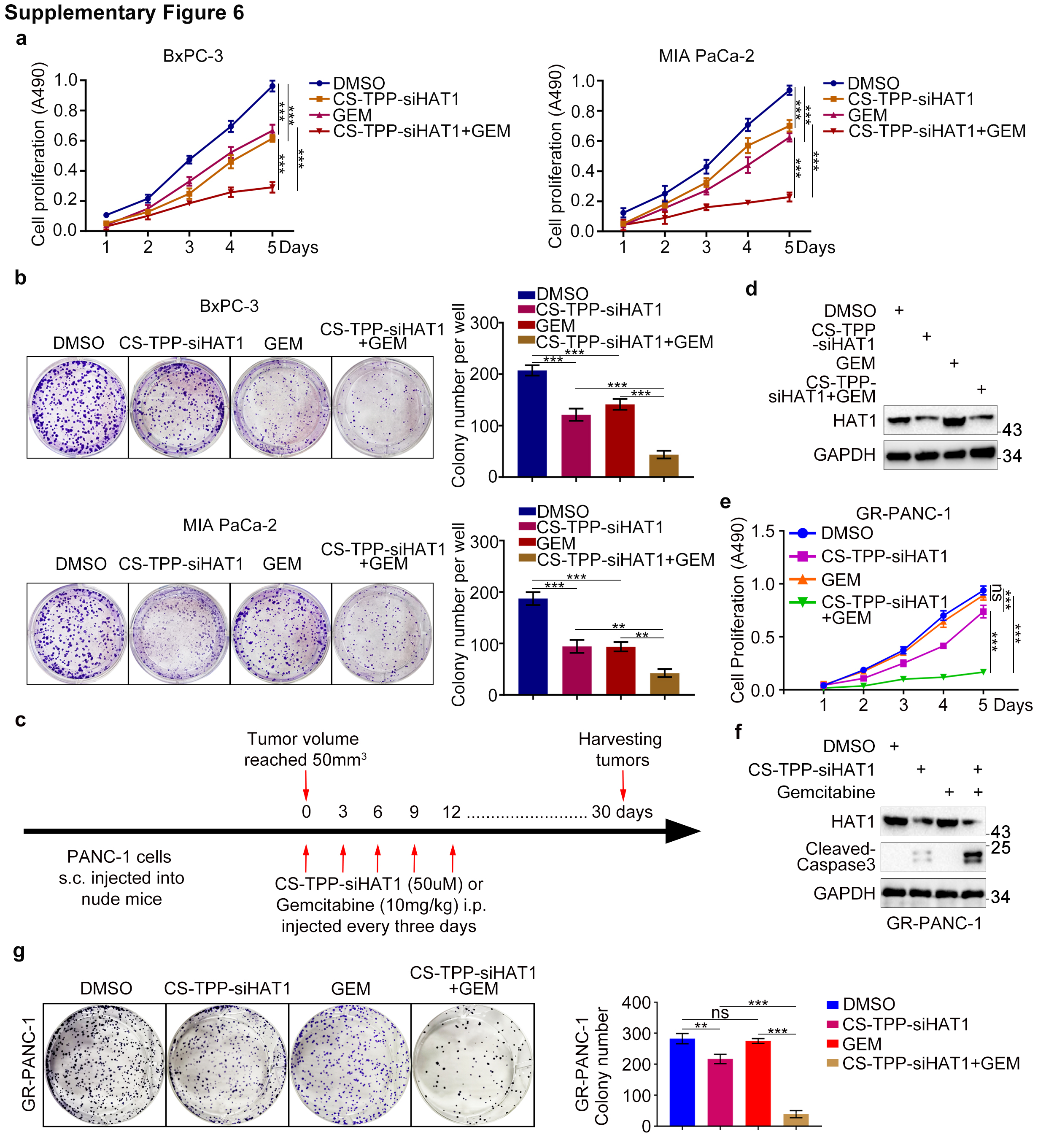

Supplement: Supplementary file 6 — supplementary figure 6 [file 41419_2021_4118_MOESM6_ESM.jpg]
