# Supplementary material for: Histone acetyltransferase 1 promotes gemcitabine resistance by regulating the PVT1/EZH2 complex in pancreatic cancer
Source: Cell Death Dis. 2021 Sep 25;12(10):878. doi: 10.1038/s41419-021-04118-4 (PMC8464605; doi:10.1038/s41419-021-04118-4)
Supplement: Supplementary file 7 — supplementary information [file 41419_2021_4118_MOESM7_ESM.docx]

**Supplementary Figure 1. a.** PANC-1, BxPC-3 and MIA PaCa-2 cells were infected with the indicated plasmids. Cells were collected for RT-qPCR and Western blotting after 48h and 72h respectively. All data were shown as mean values ± SD (n = 3). *******, P < 0.001. **b.** MTS assay detected the viability of BxPC-3 and MIA PaCa-2 cells with normal, knockdown and overexpressing HAT1 after treatment with gemcitabine. The cell viability curve showed IC50 values of gemcitabine among different groups. **c.** HPDE6-C7, PANC-1, BxPC-3 and MIA PaCa-2 cells were extracted the total proteins and RNA to conduct Western blotting and RT-PCR to analyze the expression level of HAT1. All data were shown as mean values ± SD (n=3). *******, P < 0.001. **d-f.** BxPC-3 and MIA PaCa-2 cells were infected with lentivirus expressing control or HAT1-specific shRNAs. After infecting 72h, cells were harvested and treated with gemcitabine (50nM) for MTS assay (**d**), colony formation assay (**e**) and cleaved caspase-3 activity assay (**f**). All data were shown as mean values ± SD (n = 3). *******, P < 0.001. **g.** The changes of cell morphology in different stages with gemcitabine treating. **h.** MTS assay detected the viability of PANC-1 and GR-PANC-1 cells after treatment with gemcitabine. The cell viability curve showed IC50 values of gemcitabine. **i-k.** GR-PANC-1 cells were infected with lentivirus expressing control or HAT1-specific shRNAs. After infecting 72h, cells were harvested and treated with gemcitabine (50nM) for MTS assay (**i**), colony formation assay (**j**) and cleaved caspase-3 activity assay (**k**). All data were shown as mean values ± SD (n = 3). ns, not significant; ******, P < 0.01; *******, P < 0.001. **l.** PANC-1 and GR-PANC-1 cells were extracted the total proteins and RNA to conduct Western blotting and RT-PCR to analyze the expression level of HAT1, PVT1 and EZH2. All data were shown as mean values ± SD (n=3). *****, P < 0.05; ******, P < 0.01; *******, P < 0.001.

**Supplementary Figure 2. a.** Volcano plot showed the differential expressed genes of PANC-1 cells infected by shControl or shHAT1. Differential genes compliance with the screening criteria were the red points of the genes between two blue lines. **b.** The table showed some genes directly or indirectly related to cancer were found through disease association, PVT1 was included in the table. **c.** BxPC-3 and MIA PaCa-2 cells were infected with lentivirus expressing control, HAT1-specific shRNAs, pcDNA3.1 or HAT1 plasmid. After infecting 48h, cells were harvested to conduct RT-qPCR analysis. All data were shown as mean values ± SD (n = 3). ******, P < 0.01; *******, P < 0.001. **d.** PANC-1, BxPC-3 and MIA PaCa-2 cells were infected with shControl or PVT1-specific shRNAs. After infecting 72h, cells were harvested and treated with gemcitabine (50nM) for MTS assay. All data were shown as mean values ± SD (n = 3). *******, P < 0.001. **e.** PANC-1, BxPC-3 and MIA PaCa-2 cells were infected with HAT1-specific shRNAs and PVT1 plasmid simultaneously. After infecting 72h, cells were harvested and treated with gemcitabine to perform MTS assay. All data were shown as mean values ± SD (n = 3). ns, not significant; ******, P < 0.01; *******, P < 0.001.

**Supplementary Figure 3. a** and **b.** PANC-1, BxPC-3 and MIA PaCa-2 cells were infected with shControl, HAT1-specific shRNAs or HAT1 palsmid. After infecting 72h, Western blotting was conducted to analyze the immunoblot of H4ac in PANC-1, BxPC-3 and MIA PaCa-2 cells after knocking-down (**a**) or over-expressing HAT1 (**b**). Total H4 was used for loading controls. All data were shown as mean values ± SD (n=3). *******, P < 0.001. **c.** BxPC-3 and MIA PaCa-2 cells were infected with shControl, BRD4-specific shRNAs and BRD4 overexpressing plasmid. After infecting 48h, cells were harvested to conduct RT-qPCR analysis. All data were shown as mean values ± SD (n=3). *****, P < 0.05; ******, P < 0.01; *******, P < 0.001. **d.** PANC-1 cells were treated with JQ1 (10 μM) alone, gemcitabine alone or JQ1+gemcitabine respectively. Then MTS assay was performed to detect the cell viability. All data were shown as mean values ± SD (n=3). *******, P < 0.001. **e.** PANC-1 cells were infected with shControl or BRD4-specific shRNAs. After infecting 72h, cells were harvested and treated with gemcitabine (50nM) for MTS assay. All data were shown as mean values ± SD (n=3). *******, P < 0.001.

**Supplementary Figure 4. a.** PANC-1, BxPC-3 and MIA PaCa-2 cells were infected with the indicated plasmids. Cells were collected for RT-qPCR and Western blotting after 48h and 72h respectively. All data were shown as mean values ± SD (n = 3). *******, P < 0.001.  **b.** PANC-1, BxPC-3 and MIA PaCa-2 cells were infected with shControl or EZH2-specific shRNAs. After infecting 72h, cells were harvested and treated with gemcitabine (50nM) for MTS assay. All data were shown as mean values ± SD (n=3). *******, P < 0.001. **c.** PANC-1, BxPC-3 and MIA PaCa-2 cells were infected with shControl and PVT1-specific shRNAs. After infecting 48h, cells were harvested for RT-qPCR analysis. All data were shown as mean values ± SD (n = 3). ns, not significant; *******, P < 0.001. **d.** RNA pull-down assay indicated that PVT1 bound to EZH2. **e.** PANC-1, BxPC-3 and MIA PaCa-2 cells were treated and RIP assay was performed according to the protocols of RIP kit. All data were shown as mean values ± SD (n=3). *******, P < 0.001. **f.** PANC-1, BxPC-3 and MIA PaCa-2 cells were treated with different GSK126 concentration for 3 days. PANC-1, BxPC-3 and MIA PaCa-2 cells were treated different time with 10 μM GSK126. Then cells were extracted the total proteins to conduct Western blotting. **g** and **h.** BxPC-3, MIA PaCa-2 (**g**) and GR-PANC-1 (**h**) cells were infected with HAT1-specific shRNAs. After infecting 48h, cells were harvested and treated with GSK126 (10 μM) and gemcitabine for 5 days to perform MTS assay. All data were shown as mean values ± SD (n = 3). ******, P < 0.01; *******, P < 0.001. **i.** PANC-1, BxPC-3, MIA PaCa-2 and GR-PANC-1 cells were infected with Control, EZH2-specific shRNAs and HAT1 overexpressing plasmid. After infecting 48h, cells were harvested and treated with gemcitabine for 5 days to perform MTS assay. All data were shown as mean values ± SD (n = 3). ******, P < 0.01; *******, P < 0.001.

**Supplementary Figure 5. a** and **b.** BxPC-3 and MIA PaCa-2 cells were infected with shControl, HAT1-specific shRNAs and HAT1 overexpressing plasmid. After infecting 48h, cells were harvested for RT-qPCR analysis (**b**) and 72h for Western Blotting analysis (**a**). All data were shown as mean values ± SD (n = 3). ns, not significant; ******, P < 0.01; *******, P < 0.001. **c** and **d.** The tissue microarray of pancreatic cancer (n=31) was stained with HAT1 and EZH2 respectively. The typical images of HAT1 and EZH2 were displayed (**c**). HAT1 and EZH2 IHC score of tissue microarray of pancreatic cancer was plotted the scatter diagram (**d**). **e.** PANC-1, BxPC-3 and MIA PaCa-2 cells were infected with the indicated plasmids. After 72h, Western blot analysis of exogenous HAT1 and EZH2 proteins reciprocally immunoprecipitated by anti-Flag and anti-HA in PANC-1, BxPC-3 and MIA PaCa-2 cells. Immunoblots (IB) were representative of results from three independent experiments (n = 3). **f.** PANC-1 cells were infected with HAT1-specific shRNA. After 24h, PANC-1 cells were further infected with HAT1 plasmid. After another 48h, cells were harvested and treated with MG132 (10 μM) for 16h. Then, Co-IP and Western blotting were performed by using anti-EZH2 to detect the change of UBR4 and HAT1 (n = 3). **g.** PANC-1, BxPC-3 and MIA-PaCa cells were treated with different CS-TPP-siHAT1 concentration (10 μM, 20 μM, 50 μM) for 3 days. The cells were harvested to extract RNA, and then RT-qPCR was performed to analyzed PVT1 expression level. PANC-1, BxPC-3 and MIA-PaCa cells were treated with 20 μM CS-TPP-siHAT1 for different time (0 days, 1 days, 2 days, 3 days). The cells were harvested to extract RNA, and then RT-qPCR was performed to analyzed PVT1 expression level. All data were shown as mean values ± SD (n = 3). *****, P < 0.05; ******, P < 0.01; *******, P < 0.001.

**Supplementary Figure 6. a** and **b.** BxPC-3 and MIA PaCa-2 cells were treated with CS-TPP-siHAT1 alone, gemcitabine alone or the combination for MTS assay (**a**) and clone formation assay (**b**). All data were shown as mean values ± SD (n=3). ******, P < 0.01; *******, P < 0.001. **c.** The process schematic diagram of using CS-TPP-siHAT1 and gemcitabine to treat nude mice. **d.** The tumor tissue proteins of nude mice were extracted to carry out Western blotting to analyze HAT1 expression level. **e-g.** GR-PANC-1 cells were treated with CS-TPP-siHAT1 (50 μM) alone, gemcitabine alone or the combination for MTS assay (**e**), clone formation assay (**g**) and cleaved caspase-3 activity assay (**f**). All data were shown as mean values ± SD (n=3). ns, not significant; ******, P < 0.01; *******, P < 0.001.
